# This article explains the secrecy paradox, but don’t tell anyone!

**DOI:** 10.3389/fpsyg.2026.1701598

**Published:** 2026-02-12

**Authors:** David M. Bergman

**Affiliations:** Swedish Defence University, Stockholm, Sweden

**Keywords:** critical discourse analysis, network analysis, online communities, Riksbunker, secrecy paradox, social hierarchy

## Abstract

The present study examined how the secrecy paradox—signalling privileged knowledge while at the same time concealing its content to preserve exclusivity—affected communication in an online community. Data were a comprehensive discussion thread about the existence and location of a large secret underground command centre (a “Riksbunker”) that would serve as the Swedish governments Cold War contingency plan for a potential World War III. The study used a mixed-method design combining critical discourse analysis (CDA) with citation network and community cluster analysis. Network analysis revealed a modular structure of tightly connected clusters with a small number of core users controlling the information flow. The CDA revealed six recurrent discourses that together sustained a steep hierarchy and highly stratified social order within these clusters, which emphasizes exclusivity and—ironically and paradoxically—preserves the very secrecy the thread was originally initiated to explore. One main conclusion is that the secrecy paradox can be seen not as a mere influencing factor in communicative interaction but as an emergent social practice around which social structures, identities, and norms are formed.

## Introduction

A humorous military saying is that “It’s no fun being secret if you can’t tell anyone”. It indicates that positions of proprietary knowledge are often related to higher social status. Yet, social status is not only dependent on having access to specific information but also requires making others aware that the individual is concealing that information from them (Bellman, 1981; Bergman, 2024). Being recognised as having access to information that others do not can give a strong sense of power and increased social status, but only insofar as that possession is made visible.

The paradoxical nature of secrecy in social settings often emerges from the contradictory demands to both withhold information and signal access to it. This is especially natural in some areas and discussions about topics where secrecy is naturalised and institutionally anchored like the military, intelligence community, and other national security contexts. Understanding secrecy in these settings requires moving beyond questions of what is hidden—the secret itself—to examine how secrecy operates as a resource through communicative practices for creating and maintaining social order.

### The secrets we keep, and the ones we tell

We all learn the importance of secrets at an early age. Whether it is concealing information we do not want others to have, or revealing personal information in order to connect with others. Illustrated by the common saying “Tell me your secrets, and I’ll tell you mine”.

Secrets can be both disclosed and concealed for different reasons. The concealment of information is generally concerned with avoiding detrimental effects like stigmatization, loss of status or social exclusion that could be the effect if others were to become aware of it (Liu et al., 2023). Slepian (2024) found that the most common secrets we conceal from others are often associated with romantic relationships (infidelity, hidden desires, sexual behaviour, abortions), the secrets kept with others (lies told, violations of trust), work-related (poor performance, work discontent, financial problems), but can also relate to experience of trauma as a victim or engaging in illegal activities oneself. These are all topics often related to taboo or social stigma.

Disclosing secrets can also be done for positive reasons and can hold both vitalizing and energizing effects. Many secrets hold an inherently positive capacity such as an upcoming job-promotion, a long-awaited pregnancy, or future marriage proposals. While negative secrets are generally kept for external reasons, positive secrets have shown to be more related to internal reasons (Slepian et al., 2023). Possessing secrets and keeping them hidden from others can be seen as more related to intrinsic motivation, since it is chosen voluntarily (not forced by fear of stigma), and since it relates to personal competency when individuals implement their choice of disclosure (Deci and Ryan, 1985). Keeping and voluntarily revealing personal secrets can be especially rewarding when the secrets align with something the individual values, or with their self-conception (Sheldon and Elliot, 1999).

Humans are also generally bad at keeping secrets secret. By their very nature, secrets are information meant not to be disclosed (etymology form Latin *secretum*: hidden, concealed, private). Yet we are inherently predisposed to disclose both positive and negative secrets frequently (Liu et al., 2023; Slepian, 2024). Often for social reasons. While much of the psychological literature treats secrecy in terms of individual possession and motivation, the present study draws on these findings only as background and focuses primarily on secrecy as a communicative and relational phenomenon.

Petronio and Bantz (1991) found that the added cautionary prefix “Don’t tell anyone, but …” actually increased the likelihood that the information would subsequently be passed on to others since it noted higher value. Generally, more emotionally charged information has shown to increase the likelihood of secondary social sharing since it holds intrinsic value from the discloser and elicits an emotional response from the confidant (Christophe and Rimé, 1997).

The expression “Don’t tell anyone, but …” also demonstrates the first paradoxical element regarding secrets: when someone discloses proprietary information with the implicit expectation or explicit caution that the recipient must promise not to pass it on, he violates the very confidentiality he expects others to honour. Demanding secrecy while breaking it is a contradiction in itself.

### Secrets and social positioning

The choices to sometimes conceal or reveal secrets to others make it primarily a social phenomenon. Kelly (2002) notes that the “fundamental motivation to belong may explain both why people sometimes choose to reveal and sometimes choose to keep their secrets” (p. 21). Groups are often defined by proprietary in-group information not meant to be disclosed, and individuals largely define themselves and others along these lines. Secrets have long served as a factor for how groups and associations organize themselves within our society (Shils, 1956). In his early works on secret societies, Simmel (1906, 1950) describes secrets as a constituent of public and private spheres. He points out that secrets stand in neutrality above their content and are invariant to the kinds of information that are controlled by it. In other words, being accepted into a secret society is itself often valued higher than the information the person can attain there.

This distinction is often discussed in terms of first- and second-order secrecy, where the first refers to the concealment of specific information and second to the communicative practices through which knowledge about who knows and who is excluded is determined (Petronio, 2002). Already in the early works Simmel (1906) distinguishes between secret content and social form. While first-order secrecy constitutes the substantive condition that makes secrecy meaningful, it is second-order secrecy that gives it social force. In second-order secrecy the first order—the secret itself—becomes less prevalent.

Secrets not only determine social inclusion but have also been argued to determine status and positioning in that group. Liberman and Shaw (2018) found that children start to understand secrets around the age of six but primarily that, at the same time, they learn to distinguish social connections between people based on their shared secrets. Dunbar (1991) described the disclosure of information relevant to others as “social grooming,” or with a more common expression: ‘gossip’. Secrets can function as a kind of symbolic capital when enacted and recognised in interaction (Bourdieu, 1991) and thus function as a social practice to maintain and develop ties in different groups. As Simmel (1906, 1950) pointed out, secret societies are often not completely secret but are, on the contrary, often extremely open about the existence of the organization and the fact that proprietary information is only given to insiders. Sometimes almost flaunting the concealment of information to add value for those included.

Revelations of secrets are more likely to be directed towards upper level in hierarchies, indicating that they are connected to achieving a position of higher social standing (Youvetich and Drigotas, 1999). Derlega and Grzelak (1979) described five motives for revealing secrets to others: self-clarification, social validation, relationship development, social control, and expression. How an individual manages secrecy will often determine their sense of self and the kinds of relationships they have with others.

The second paradoxical element of secrecy is that disclosure can grant inclusion and a raise in social status but at the same time erodes both the secret and the individual’s uniqueness: an individual must disclose proprietary information for inclusion and to attain status in different social groups, but once revealed, the secret will no longer be secret and the more the individual discloses, the less special he becomes.

Regarding the present study, the paradoxical effects of secrecy are primarily located at the level of second-order secrecy, as this is the locus where secrecy becomes socially productive. Ontologically, this study conceptualises secrecy as a relational and practice-based phenomenon that comes into being through communicative interaction in social settings, rather than as a fixed object, an attribute of information, or an individual mental state. This understanding builds on classical work by Simmel (1906, 1950), who conceptualises secrecy as a social form structured through relations of inclusion and exclusion; secrecy exists between people, not inside information. From this perspective, the secrecy paradox can be understood as an emergent effect of communicative practices rather than as a contradiction rooted in the content of secrets themselves.

### The secrecy paradox and its potential resolve

Bellman (1981) was the first known researcher to coin the term “paradox of secrecy” to summarize the contradictory elements described above. He noted that since secrets are inherently meant not to be disclosed, doing so often violates the basic principle of the secret. And when the secret has been revealed it will no longer be a secret.

These effects are a social equivalent to the Liar’s paradox (Schurz, 2012); when the liar states that he is always lying he is in fact telling the truth, which means the liar just lied. Both paradoxes expose fundamental tensions in systems governed by self-reference that point to systematic limitations. But what differentiates the two is that the liar’s paradox will collapse under its own logical contradiction (see, e.g., classical works: Kripke, 1975; Tarski, 1944), while the secrecy paradox is a functional paradox that ‘only’ undermines its own social utility. This means that the secrecy paradox is one that can theoretically be practically managed and stabilised through the practises of social interaction.

Friedrich Hegel (1969) pointed out that in order to understand paradoxes we have to abandon our conviction that only separate ‘no’ or ‘yes’ are possible answers. His idea was basically that no unilateral part is “true” and that a deeper examination of the elements of the paradox can reveal insight into the concept at stake. Simplified, we need to accept that the answer to a paradox is not simple. In order to understand ‘true contradictions’ we have to investigate how the contradictory terms interact (d’Agostini and Ficara, 2022).

This functional interaction of contradictory elements within a paradox is often referred to as a resolution, or that the paradox has been resolved (e.g., Smith and Lewis, 2011). This is not used literally to imply that the secrecy paradox is solved logically or eliminated. Rather, that the paradox is practically managed and sustained over time. In his classical works Hegel (1969) uses the German word “*Aufhebung*” (p. xvii) to describe a process where something is both preserved and changed through dialectical interaction: the paradox is stabilised and rendered functional in practice.

To exemplify we can more closely examine secrecy as a relational phenomenon in social settings. The previously mentioned “Don’t tell anyone, but …” statement used to describe the paradox of secrecy (Bellman, 1981; Petronio and Bantz, 1991) can be viewed as inherently self-contradictory. But if we re-examine the contradictory terms more closely we find that the statement about disclosure can also be viewed as an act of legitimate informing rather than as a breach of exposing, thereby transferring “Don’t tell anyone” from its literal meaning, to instead mean “Don’t tell anyone not socially included”. The inductee is therefore not only a recipient of information but also trusted to maintain the integrity of the secrecy and treat others as those meant to be excluded, or as prospects considered for inclusion on the same terms. Consequently, we can see a possible resolution to the paradox: the individual will disclose as much information as needed to as many individuals necessary to achieve the maximum effect possible in social inclusion and status, yet not so many that it violates the integrity of the group or devalues the information required for the increase in social status to occur. Thus, the contradictions of the secrecy paradox can theoretically coexist and interact in a logical way where the paradox is rendered socially stable.

Despite the fact that individual secrets are a notable branch of research [for a good overview see, e.g., Kelly (2002) and Slepian (2024)], the contradictory elements of secrecy for social positioning have received little attention. Existing studies have often focused more on rationalism than empirical studies (e.g., Bellman, 1981), leaving these functions of the secrecy paradox relatively unexplored. A neighbouring field of research is that of conspiracy theory research (for a good overview see, e.g., Demata, 2025) that also focuses primarily on second-order secrecy. But the present analysis is not primarily concerned with why participants believe certain claims, rather with how they relate to each other through communicative interaction in social settings. Thus, the primary aim of the present study was to empirically examine the presence and function of the secrecy paradox within interpersonal communication regarding a highly secretive topic.

## The present study

The present study examines how secrecy and the secrecy paradox is enacted and sustained through communicative practices in an online discussion forum centred on a highly secretive and institutionally protected topic: The existence and location of a large secret underground command centre (a “Riksbunker”) for the Swedish government in times of war. A subject that is arguably one of the biggest secrets a country, an organization or an individual can possess. During the Cold War era Sweden had a strict non-alignment policy, and a clearly defensive strategy aimed at using fortified facilities. The discussions about such facilities have been heavily influenced by the declassification of the West German “Regierungsbunker” in Marienthal (Diester, 2009), and the Danish government’s underground command centre Regan West, south of Aalborg (Pedersen and Pedersen, 2022). But Sweden’s Cold War plans for a potential World War III have never been declassified, making it a topic related to extreme secrecy and thus intense interest, discussion, and speculation. Rather than analysing the factual accuracy of claims (purposefully avoided) the study focuses on how secrecy itself functions and shape communicative interaction in social settings.

The overarching aim is to understand how contradictory demands of disclosure and concealment are practically managed over time, and how this management contributes to the emergence and reproduction of social structure.

Research Question 1: *How does the secrecy paradox manifest in communicative practices within an online community centred on a highly secretive topic?*

Research Question 2: *How do discursive strategies contribute to the emergence and reproduction of boundaries, hierarchy and status within the community?*

## Method

### Data

The data were collected from the public web forum flashback.net in January of 2025. Flashback Forum is Sweden’s largest user-driven open discussion platform, with an emphasis on free speech. Specifically, the empirical material consisted of a single, long-running discussion thread on the forum about the Riksbunker initiated on 31 July 2008. Meaning that at the time of data collection, the discussion thread had been active for roughly 16.5 years.

The choice of empirical data was made since it presents a comprehensive material (further described below) potentially rich in meta-communication relevant to the secrecy paradox. Selection of open access material available to the general public also increased scientific transparency, verifiability, and reproducibility.

### Design

A mixed-method design was chosen, combining both quantitative and qualitative methods. The multifaceted approach was selected since it offered the opportunity to both explore trends and structures in the extensive data, as well as the in-depth nuances of individual communication.

The analytical steps were designed to be analytically integrated and mutually reinforcing rather than independently triangulated to test convergent validity. Descriptive statistics was used to establish distribution, equality and persistent nature of the discussion. Network analysis then operationalised this by identifying core structures and patterns of recognition. Building on these structural findings, the Critical Discourse Analysis examined how communicative practices enact and reproduce these patterns over time. In this way, the qualitative and quantitative analyses address different aspects of the same phenomenon, with structure and discourse informing each other.

### Analysis

The quantitative element consisted of a descriptive statistical analysis and the construction of a citation network with cluster detection. To get an initial overview of the vast dataset, a series of descriptive analyses was performed to summarize key characteristics of the forum discussion, such as user participation, message lengths, patterns, and distributional trends. The Gini coefficient was computed to quantify inequality in posting frequency among users, as well as parameters related to longitudinal changes.

To further analyse the relational structure of user interaction, a directed citation network was constructed. By examining how users referred to and quoted other users, it was possible to construct the directionality and temporal order of the interaction. To reduce noise and improve interpretability, the analytical threshold was set to the 20 most-cited users (determined by in-degree centrality) who collectively constituted the thread’s internal core structure. Finally, in order to detect underlying structural subgroups, a greedy modularity maximization algorithm was used to detect non-overlapping clusters and nodes by comparing internal versus external citation density (Clauset et al., 2004). The stable core structure persisted across alternative thresholds (e.g., top 30 users). Including a larger number of nodes did not add anything substantiative but primarily reduced interpretability by introducing a tail of sparsely connected actors.

The qualitative element consisted of a Critical Discourse Analysis (CDA) (van Dijk, 2008). CDA was selected since it sees the discourse—language use in speech and writing—as a form of “social practice,” making it suitable for communication related to status, identity, and power (Fairclough, 2013). Although initially adopted for communication in news media and political communication, the framework of van Dijk (2008) remains foundational. Subsequent work has adapted CDA to address the specific communicative forums such as online and social media platforms (e.g., Bouvier and Machin, 2018; Abbas et al., 2025) since the analytical frameworks is useful for both linguistic phenomena and the interactive, user-generated nature of online dialogue. It has also been specifically recommended for mixed-method design, and used together with quantitative methods since it minimizes the risk of “cherry picking” (choosing the examples which best fit the researchers’ assumptions), and instead adds interpretive depth to frequency-based findings (Wodak and Meyer, 2009). Coding was conducted by a single researcher.

Identification was made of posts that held codes relevant to the research question. For example, the quote below was selected since it held several relevant codes in how pronouns were used (‘we’ who know, ‘those’ who do not), how information was elevated (‘mystery of the century’), and how potential access to sensitive information was framed.

“This discussion is an incentive for those who don’t know. Myself and a number of others know. Of course, we would never dream of posting it on Flashback! Through hard work /…/ those with inferential skills can at best get on the track of the UE (Urban Exploration, authors note) mystery of the century. Unfortunately, there are no free lunches! This is a very difficult task so good luck. Start from the facts and ask yourself the right questions. If you’re lucky, you’ll find the answers. The solution is out there!” *Thread post #349, posted 2008-08-24, 00:44.*

The CDA was structured according to Fairclough’s (2013) three-dimensional model (micro, meso, and macro). The first step (micro) included a close reading and identification of vocabulary, metaphors, modality, and pronoun use related to secrecy and social positioning. This included words and phrases such as “We who know”, “big brother”, “take it via PM (private message, authors note)”, “disinformation”, “charlatans”, and “I think you [username] understand what I’m looking for…”. On the second level (meso), alternation was made between inductively identifying patterns and deductively testing them against the secrecy paradox, which facilitated the identification of several discursive strategies. For example, the recurrent mentioning of private messages was interpreted not primarily to inform the explicit recipient that he had a waiting message, but to inform everyone else that there was information they would not be entrusted with. Hence, it was coded to the ‘gatekeeping’ discourse. In the third (macro) level, the findings were summarized into the sociocultural practice, where the identified discourses were embedded. These patterns were subsequently refined as the work progressed. Posts could belong to more than one potential discourse and often had multiple code elements, as the example quote illustrates.

Finally, it was possible to summarize the discourse patterns and draw conclusions. Since the secrecy paradox was inherently present in various and different forms in all identified discourses, an attempt was made to summarize and resolve each sub-paradox in the respective discourses. These proposed resolutions were observed emergent patterns that participants used to navigate the contradictory demands by discursive practices and how the paradox was practically managed and sustained over time in a balanced way without collapsing. In this regard, resolution does not imply that the sub-paradox was logically solved or eliminated but how it was stabilized and rendered functional in social practice.

Analytically, the study distinguishes between individual-level communicative actions and collective-level discursive outcomes. While the analysis examines how individual participants deploy specific linguistic strategies, the identified discourses are not treated as intentional group norms or coordinated strategies but rather emergent patterns from repeated social interaction over time, producing relatively stable structures of hierarchy and inclusion without requiring central coordination.

The empirical material was in Swedish. The analysis was performed in the original language and the results then translated to English by the author. Since the analysis focuses on discursive functions rather than idiomatic or culturally specific expressions, translation into English did not pose any substantial challenges. Example statements to illustrate, superfluous are paraphrased linguistic patterns rather than reproduced direct quotations for ethical reasons.

A few words on anomalies and outliers. In the discussion thread, there are explicit accusations of off-topic discussions, provocative or manipulative posts (trolling), as well as statements regarding the possible presence of authorities and/or foreign powers in the discussion, making deliberately leading or misleading posts. This was identified as a possible threat to validity. An awareness of this was maintained throughout the analysis. Although there are users and posts that regardless of the reason did indeed show such possibilities, these were most often those who offered nothing substantial in relation to the research question and were automatically omitted for this reason.

### Ethical issues

The project and its procedures were approved by the Swedish Ethical Review Authority.

Measures were taken throughout the project to maintain the anonymity of all users on the forum with concern for personal integrity. Most users used semi-revealing pseudonyms for their username (reasons elaborated below) making identification of their personal identities difficult but not impossible. Consequently, any reference to usernames and direct quotations has been avoided as far as possible in order to prevent possible identification of specific individuals.

Since the topic of the discussion was related to matters of national security, several precautionary steps were taken to minimize any reference to potentially sensitive information. Although the empirical material speculates about secret government facilities the analysis is not concerned with the factual accuracy of claims. Instead, the focus is on how secrecy is communicatively enacted, signalled, and negotiated in interaction, regardless of whether the underlying claims are true, false, or unverifiable. The chosen scientific method inherently mitigated security concerns by focusing primarily on *how* the users communicated rather than *what* they were discussing.

In addition, quotes and examples in the text that could potentially touch on sensitive information were included in the analysis but intentionally left out of the final article. Focus has been on the individuals’ discussions regarding top secret information, but any indicators of their accuracy in those discussions have been avoided.

### AI usage disclosure

This article was prepared with limited assistance from AI-based tools. Specifically, the extraction and initial structuring of forum data were performed using Octoparse v8.7.2.090415. In addition, a large language model–based generative AI system was used as a supportive tool to assist in organising and summarising the large dataset into manageable formats. AI-based tools were also used to support the organisation and calculation of statistics.

Most importantly, generative AI was not employed in the subsequent analysis, interpretation of results, or the authoring of the article’s main text. All theoretical framing, analytical coding, interpretation, and conclusions are the responsibility of the author. The AI support was restricted to routine processing and similar technical tasks. Its outputs were systematically reviewed, verified, and refined by the author to ensure academic integrity, validity, and originality.

## Results

### Descriptive statistics

The thread has 502 unique users, with a total of 7,644 posts (over 8,000 had been posted but some were deleted by moderators over the years). Individual posts have a mean of 128 words and the entire thread has a total of 980,092 words. For reference, that is double the length of the Lord of the Rings trilogy, with 481,103 words in total [See Tolkien (2009a, 2009b, 2009c)].

Regarding distribution, the 20 most active users are responsible for nearly half (49.01%) of all the posts in the thread. The Gini-coefficient was 0.788, indicating a high level of inequality and a highly centralized discussion structure, with the bottom half of users contributing very little to the overall discussion. The posts from the top users are also longer than the others, with 137 versus 108 words per post, indicating more substantial content.

The analysis of the forum thread over its 16.5-year lifespan (2008–2025) reveals that the discussion can be roughly categorized into three phases. In the initial phase (2008–2012), the interactions focus more on open information exchange, with an emphasis on locating facilities and determining their functionality within the wartime contingency plans. During the second phase (2013–2018), the interactions evolve toward more technical and functional interpretations. The later phase (2019–2025), sees a continued discussion but with a general decline in participation and an increase in risk-conscious communication framed within a broader security policy debate. The secrecy paradox is increasingly salient over all three phases.

### Citation network with cluster detection

**Figure 1:**
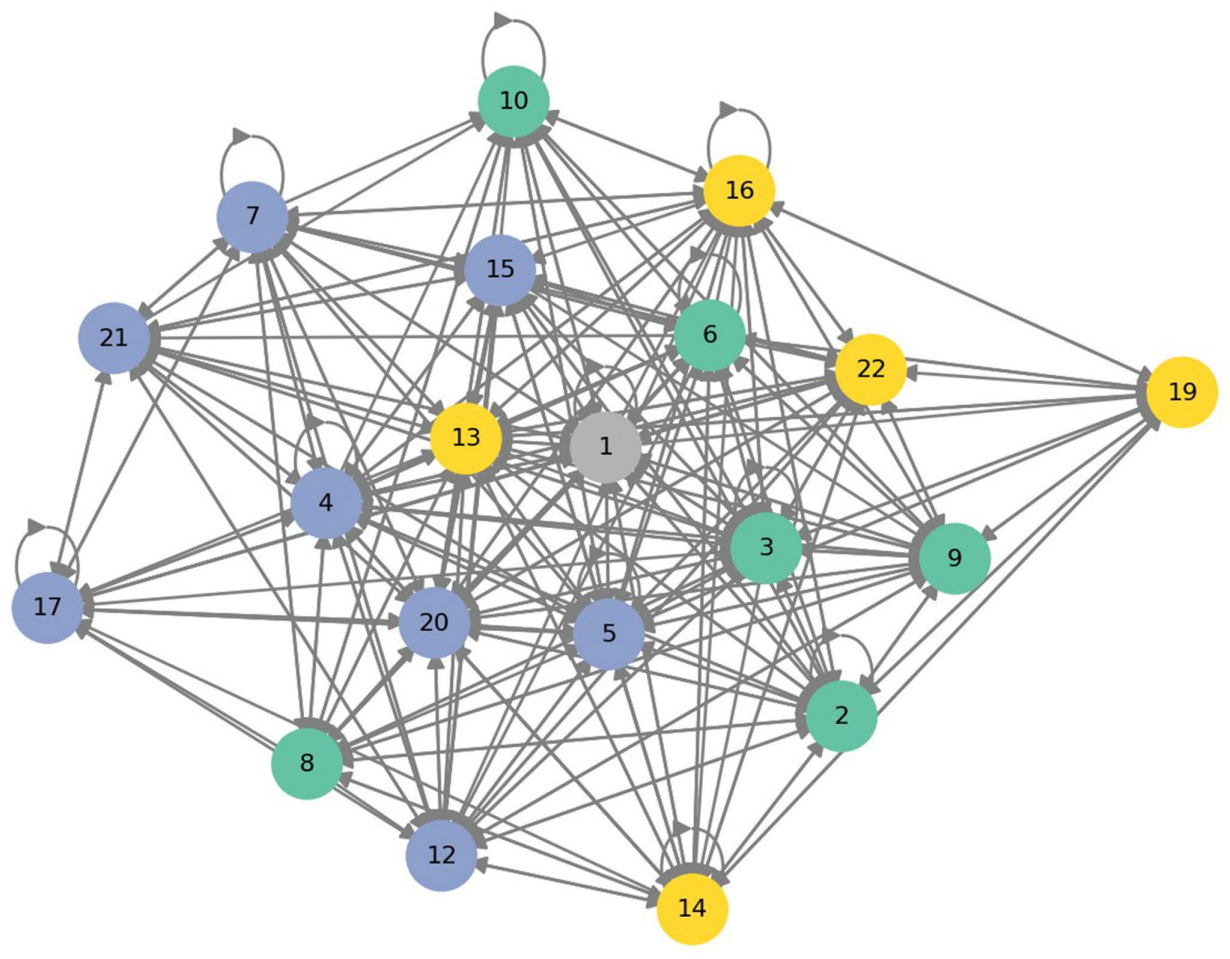
Citation network structure and community clusters.

A directed citation network and identified clusters can be seen in Figure 1. In the visualization (Figure 1) nodes are labelled with numerical identifiers based on their number of posts (with 1 the most active and so on), node colours indicate cluster affiliation, and arrows indicate the direction of the quotes and mentions (including circular self-citations). Note that the 20 most cited and 20 most active users are roughly but not exactly the same (ID 1–10 are exactly the same). The positioning on the x- and y-axis in the figure reflect relational proximity, where users who cite each other frequently are placed closer together.

The following clusters were identified.

#### Green cluster—core contributors

These comprise the top ranked users who form the discursive nucleus of the thread. Their posts are information-rich and often cited by others, positioning them as interpretative authorities. This included the number 1 user in the thread marked in grey at the centre of the figure.

#### Blue cluster—peripheral contributors

These users interact frequently with the core contributors but most often on engaged elaboration, such as clarifications and verifications, supporting the core contributors but still displaying a strong intra-cluster citation amongst themselves.

#### Yellow cluster—specialized contributors

These users primarily focus on specific sub-topics with lower inter-cluster connectivity, making them stand out as active in the discussion yet more topically bounded and less connected to others.

The citation network and its clusters demonstrate that the discussion is not random or evenly distributed but shows cohesive subgroups that also form a hierarchical structure. It illustrates that from a dataset of 502 unique users, there are 7 + 8 users who form the primary and secondary nucleus of the thread. The results also demonstrate that this is not solely a function of participation volume, but that the structure has been formed and reinforced through reciprocal recognition and community validation, creating a steep hierarchy and highly stratified social order.

### Critical discourse analysis

A total of 3,245 posts were recognized as containing at least one element of code related to the research question. From these posts a total of 3,858 discourse-level instances were identified (a post could be associated with more than one discourse category). Within these discourse-level instances a more fine-grained coding of linguistic markers and communicative statements resulted in a total of 8,422 code occurrences. The analysis identified six discourse patterns presented in Table 1. The example statements and linguistic markers in the table are illustrative and paraphrased. They are intended to demonstrate typical discursive forms rather than to reproduce empirical quotations. This approach was chosen to maintain ethical safeguards while maintaining analytical transparency.

**Table 1:** Overview of the distribution and frequency of the discursive patterns identified in the critical discourse analysis together with example markers and statements. In a CDA frequency should not be interpreted as a proxy for explanatory value. Some of the discourses, most notably scarcity, are analytically central despite low frequency.

Discourse	Code occurrences (n)	Share of total codes (%)	Example of linguistic markers and example statements	Primary function in relation to the secrecy paradox
Pseudonymity	4,825	57.3	References to past roles or affiliations (“having worked in”), appeals to experience (“those who have been around long enough”)	Identity signalling and credibility construction under conditions of risk
Hinting	2,326	27.6	Strategic vagueness (“those who know, know”), ellipses (…) and withholding (“I cannot go into details here”)	Signalling privileged knowledge while preserving secrecy
In−/out-grouping	821	9.7	Pronoun contrasts (“we who understand” vs. “those still looking”), presuppositions (“as you already know”)	Boundary maintenance and epistemic differentiation
Loyalty and risk	249	3.0	Moral caution (“do not give the enemy pieces of the puzzle”), legal limitations (“certain things are still protected”)	Moral and legal self-justification for partial disclosure
Gatekeeping	133	1.6	Conditional access (“if you had the right background”), references to private message (PM) channels (“Let us take it in PM”)	Regulation of access to information and group membership
Scarcity	68	0.8	Valuation markers (“the truly important aspect”), hierarchy cues (“I and a number of others know”)	Status construction through rarity and exclusivity of knowledge
Total	8,422	100.0		

### Pseudonymity

The first discourse pattern is related to how the users have identified themselves and built a legend around a form of semi-anonymity (pseudonymity). They present themselves almost exclusively by pseudonyms (exceptions exist but are few), with semi-disclosing legends that evoke rank, roles, or institutional affiliations that offer some information about their background but in very vague terms. Disclosure of personal information has most often been done in connection to specific claims, either voluntary or when questioned by others how they ‘know’ certain things. Users who participate in such disclosures were more likely to attain a rise in social status. This practice is common on the forum as whole, but in this specific case, it is done because of the morally questionable status of the discussions, and the risk that “big brother” (i.e., Regular Police Authority and Swedish Security Services) is watching. In subsequent posts, these pseudonyms have often functioned as pre-established warrants of credibility, thus reducing the need to substantiate new claims.

Relating to the secrecy paradox, this was identified as an identity signalling tool. The paradoxical element is that users want to conceal their exact identities to avoid detrimental consequences, but at the same time they sometimes need to disclose personal information (elaborated in other discourses) in order to build credibility around their legends and attain higher social status. A proposed paradox resolution to how the paradox is managed in practice is that users will disclose as much information as needed to maximize credibility while minimising exposure.

### Hinting

This discourse contains strategic vagueness regarding the access to information. This is done through meta-comments and explicitly withholding further information. The communication positions the sender as someone having privileged access to secret information but at the same time preserving the value of that access by refraining from elaborating in more explicit terms. This discourse is close to the classical operationalization of the secrecy paradox (Bellman, 1981). Given the spread of statements, it is unlikely that everyone will have the correct insight they implicitly claim to have.

Relating to the secrecy paradox, this was identified as the primary social practice of enactment where users publicly signal having privileged insight. A proposed paradox resolution to how the paradox is managed in practice is that individuals will make comments about their knowledge of secrecy as revealing as possible, but without explicitly disclosing it.

### In/out-grouping

This discourse concerns the social categories of participants. The purpose was the linguistic drawing of social and epistemic boundaries in order to reinforce group identity. This discourse was marked heavily by pronouns such as ‘we’ who already know and ‘they’ who are still looking, and presuppositions such as ‘you understand’, illustrating shared insider knowledge. This demonstrates users positioning themselves inside an inner circle while at the same time labelling others as outsiders. Interestingly, the praises within the ingroup sometimes border on back-handed compliments with an implicit meta-level, where the person giving the praise does so in a way that indicates that they are one of the few who have sufficient insight to assess the recipient’s level of knowledge. This indicates a social struggle, even in the core of top users. In other words, some of them were trying to raise their own status by praising others who were more informed. The positioning continuously reinforces epistemological boundaries and legitimizes differential access to information.

Relating to the secrecy paradox, this discourse was identified to establish social boundaries that make knowledge of secrecy meaningful. It also supports the social structure in which the secrecy paradox functions by excluding more individuals than it includes (if too many are included then the ingroup loses its value). A proposed paradox resolution to how the paradox is managed in practice is that the social grouping will primarily function in a self-regulatory manner to uphold the inherent inequity of the structure, allowing enough individuals needed to uphold the ingroup but still withholding the majority in order not to undermine status.

### Loyalty and risk

This discourse encompasses the security concerns regarding the facilities that are the topics of discussion, as well as the individuals balancing their curiosity against security concerns, loyalty obligations, and the legal obstacles to open discussion. The discourse regarding loyalty and risk includes arguments both for and against the discussions at the same time. From one perspective, individuals are cautioning about not being too explicit in the discussion, often emphasizing the need for secrecy, up to openly questioning the thread’s continued existence. Legitimizing non-concealment into a moral and responsible act thus legitimizes the structure of the paradox. At the same time, they downplay the need for secrecy (other nations have declassified similar facilities), and reason for a detrimental minimization of possible consequences (the Russians already know everything, right!?), contending that what can be disclosed should be, thus still sanctioning disclosure under specific circumstances. It is important to emphasize that these are not arguments exchanged between different fractions in the thread with conflicting opinions, but arguments which are equally ‘true’ at the same time, and exchanged within the group by the same individuals. A puzzle-analogy is repeatedly used both to discourage participants to share what they know (the enemy is looking for pieces of the puzzle, keep your part to yourself) and to encourage them to gather more information and share what they know (gather more pieces so we can solve the puzzle together). A similar contradiction can be seen in discussions regarding the legal aspects, both warning about the serious legal violation one is potentially committing while at the same time looking for loopholes in the existing framework that can be interpreted to one’s advantage. In this regard, the legal considerations are not made in order to determine whether or not they should discuss classified information in an online discussion forum, but rather to went legitimate concern about the unsuitability of such actions while at the same time trying to find a justification for an activity they were already involved in.

The paradoxical element identified was that a high degree of secrecy is central to the lure of exploring the subject, but at the same time, individuals must find some sort of self-justification in order to cross moral and legal boundaries. A proposed paradox resolution to how the paradox is managed in practice is that individuals will voice as much concern as possible over the unlawfulness of the actions in order to minimize guilt and shame, but only as long as they can maintain a justification as to why it is still acceptable in the specific context.

### Gatekeeping

This discourse focuses on the procedures that either allow or deny access to the ingroup or sensitive information. This includes formal credentials like military service, ranks, and units, as well as the absence of them: “if you had done your conscript service …”. The gatekeeping-discourse also includes the co-existence of open and private communication channels and their function; for example, the open shift to private messages to certain actors or an open invitation to respond privately. Openly communicating about concealed information functions both as a barrier for sensitive information that should not be disclosed in open channels but also primarily as a gatekeeper against outsiders by indirectly informing them of the existence of concealed information that they are not entitled to. The discourse also includes stated procedural barriers (searches in archives, performed fieldwork …) and the hardships required for exclusive knowledge that (maybe) could lead to acceptance in the inner circle.

Relating to the secrecy paradox, this discourse was identified as implementing practical controls of information flow, both between and within the social groupings. A proposed resolution in which the paradox is managed in practice is that the gatekeeping function will regulate the (non)disclosure of information, ensuring that it happens selectively in a way that both preserves secrecy and enhances the symbolical position of the speaker.

### Scarcity

This discourse concerns the social and symbolic value of information. It is rare in the material but still considered to be central since it overtly positions certain information as scarce and status-enhancing. Not much information in the discussion holds such value. But certain pieces of knowledge are presented not only as *facts* but as *rare possessions*, accessible only to a select few. The codes in this discourse are centred around what is ‘truly important’ for the topic and which thereby elevate the social status of the speaker. For example, comments about ‘which’ of the facilities is most interesting, implicitly indicate both overall knowledge of all facilities as well as the specific insight to determine that one of them is most interesting for the current discussion. The function in interaction is to achieve an elevation in status, steer the conversation to what is deemed more important and, as a negotiation tool, disclose certain parts to reinforce the credibility of themselves or a certain argument (linking to other discourses).

The paradoxical element identified in the discourse is the status as a value-building component; the rarer the information the more valuable it becomes, but if it were to be openly disclosed its scarcity (and its symbolic value) would disappear. A proposed paradox resolution to how the paradox is managed in practice is that the possessor of valuable information will signal its existence but protect its release as much as possible.

### Summary—critical discourse analysis

To summarize, at the macro level these discourses together shape and uphold a communicative order with natural epistemological inequality and a steep hierarchical order amongst the users. The secrecy paradox becomes visible not as a friction in communication but as a vital element in a status-maintaining function that serves both the individuals’ sense of identity as well as group-level norm enforcement. The discourses jointly sustain a system centred on the secrecy paradox, in which knowledge is signalled to enhance status and social positioning while explicit details are withheld to preserve its value. This means that the discussion thread aimed at discovering classified facilities has a discourse that prevents information about such facilities from being disclosed. Within such a framework, the discussion thread shows the potential to continue indefinitely since it has no realistically attainable end-state.

## Discussion

### Emergent social practice, not a by-product

The combined findings of the quantitative structural interaction patterns and the qualitative Critical Discourse Analysis show a multi-layered communicative ecosystem where the secrecy paradox is central. The citation network and the community clusters show that information is centred around distinct core communities, each with relatively high internal cohesion and limited inter-community exchange. The network analysis provides important contextual conditions for understanding why these discourse patterns emerge. In a setting characterised by uneven information flow and clustered interaction, secrecy-related discourses become effective means of signalling epistemic position without full disclosure. Of more than 500 unique users, the primary and secondary core clusters of seven and eight top users, respectively, make a steeply hierarchical structure within the discussion thread. Although every discussion arguably has a most frequent contributor, the results demonstrate that the top contributors are also the social core. The use of mixed design helped illustrate that the observed hierarchical structure was not merely a by-product of participation volume but actively reproduced through discursive practices related to secrecy and status.

The CDA identified six discourses that, in different ways, confirm and elaborate on the network dynamic, and relate directly to the secrecy paradox. The thread establishes a clear social categorization formed around a small number of core users (in/out-grouping), where social structuring is built through non-disclosing disclosures (Hinting) of special insight and sensitive information (Scarcity), which is primarily reserved for the inner circle (Gatekeeping). The balance between curiosity and security (Loyalty and risk) functions both as an enabler and limiter for the morally questionable discussion, which is also a major factor related to how the individuals identify and present themselves to others (Pseudonymity). The CDA sheds light on how the identified network hierarchies are reproduced over time; Discursive practices such as hinting and gatekeeping contribute to maintaining epistemic boundaries and reinforcing the authority of central core clusters.

Altogether, this puts a somewhat new perspective on the secrecy paradox. Previous research (e.g., Bellman, 1981; Kelly, 2002) has framed secrecy as vital to social positioning but primarily as a motivating factor. The findings of the current study indicate that the secrecy paradox has the capacity to function not only as a contributing factor but as an emergent social practice to how communicative discourse, identities, and norms are formed. This expands the knowledge from a conceptual description of the dilemmas of secrecy to a potential model for how a community self-organizes.

### Self-sustainability and an infinity discussion

One notable effect, believed not to have been previously discussed, is that discourses resting heavily on the secrecy paradox can become self-sustaining, giving the discussions the capacity to continue indefinitely. The social positioning ensures that outsiders will try to prove themselves worthy of insider status and the core users will utilize gatekeeping and restriction of information to keep the inner circle small enough not to devalue their own positions. The more valuable the secret is perceived to be, the greater incentive to restrict it—thus reinforcing the paradox in a self-sustainable way. Since value depends on scarcity, holders of information have a stronger incentive not to fully reveal it since full disclosure would destroy its currency and thereby their current or potential social status. This expands the classical views of secrecy as symbolic capital (i.e., Bourdieu, 1991) to the present system where scarcity is both formed and preserved in an interdependent way.

This is easier to grasp if we exemplify: if someone with critical insight were to post all of the sought-after classified information in full, it would instantly cease to be a secret, and they would lose the very exclusivity that made them (and others) special. Potentially, this has the capacity to collapse the entire social structure, which is arguably not what those taking part in the discussions would want. Users thus have more incentives to uphold the norms of the discussion than to try and reach any end-state or expose a definitive answer in terms of the first-order secrets. But even in a case of over-disclosure, the system has the inherent capacity to handle such incidents. Those in the out-group would not necessarily have enough insight to value the correctness of the information, and other core contributors of the in-group would have the opportunity to, as seen in the Scarcity discourse, devalue any such over-claims by stating that those were not the ‘real’ secrets. Stating that they still held the knowledge to what was ‘truly important’, thereby rebalancing the social structure and reducing the over-sharer to a lower social status.

Given this logic, it is not surprising that the discussion thread has continued to live on for 16.5 years. The thread launched to expose one of the state’s biggest secrets has—ironically and paradoxically—evolved into a self-regulating system that counteracts risk that the full revelation will likely occur. But it also means that it holds the capacity to continue indefinitely.

### Limitations

This study adopts a single-case, theory-driven design from a culturally and institutionally specific online forum which provides longitudinal and analytical depth but limits empirical generalisation. The qualitative analysis and single-researcher coding may also introduce a degree of selection bias. This risk was mitigated by the mixed-method design, longitudinal scope of the material and the focus on discursive patterns rather than isolated contributions. The absence of participant demographic data also reduces generalization.

Taken together, these limitations suggest that the findings should be interpreted as an analytically grounded account of how the secrecy paradox can be enacted and sustained in a specific digitally mediated social setting, rather than as a comprehensive or representative description of online secrecy practices more broadly. While similar dynamics may be observed elsewhere, their specific configuration should be understood as context-dependent.

### Future research

The present study used an integrated approach with CDA and a network-structural examination of the secrecy paradox in an online community. The findings demonstrate that the secrecy paradox can be seen not only as a factor but as an emergent social practice; however, several avenues remain for further research.

First, although the empirical material to the present study is comprehensive it is still limited to a single thematically focused forum-thread. It is unknown whether the identified discourses form generalizable social practices of secrecy that transcend social, cultural, and technical barriers. Future research could conduct similar comparative studies across other communities (both online and offline) to examine whether the same discursive practices emerge in other settings as well. For example, conspiracy theory research or other areas where secrecy is naturalised and institutionally anchored (e.g., medical and legal areas).

Secondly, other forms of analysis could offer additional insight into the longitudinal development or functioning around certain pratices. For example, regarding the use of identity building and how credibility is formed. Although the current study identifies pseudonymity as something that contributes to in-group trust, other research has shown that although anonymity is often used to promote free speech and avoid detrimental consequences, it can also lower accountability for wrongdoers and cause substantial friction (trolling) in online communication (Véliz, 2019).

Third, further focus on socio-political events could offer greater insight into the calibration of the paradox to contextual factors. Although the current study found the secrecy paradox prevalent over time there was a marked decrease in participation and a noticeable shift in discourse, with explicit mentions to the security situation (most noticeably the Russo-Ukrainian war). A more thorough cross-case or more longitudinal design could potentially reveal how discursive strategies and social structures are potentially recalibrated under changing risk-perceptions.

## Data Availability

The original contributions presented in the study are included in the article/supplementary material, further inquiries can be directed to the corresponding author.
